# Metabolic adaption of cancer cells toward autophagy: Is there a role for ER-phagy?

**DOI:** 10.3389/fmolb.2022.930223

**Published:** 2022-08-03

**Authors:** Debora Gentile, Marianna Esposito, Paolo Grumati

**Affiliations:** ^1^ Telethon Institute of Genetics and Medicine (TIGEM), Naples, Italy; ^2^ Scuola Superiore Meridionale, Naples, Italy; ^3^ Department of Clinical Medicine and Surgery, Federico II University, Naples, Italy

**Keywords:** cancer, autophagy, ER-phagy, hypoxia, ER stress, UPR, endoplasmic reticulum

## Abstract

Autophagy is an evolutionary conserved catabolic pathway that uses a unique double-membrane vesicle, called autophagosome, to sequester cytosolic components, deliver them to lysosomes and recycle amino-acids. Essentially, autophagy acts as a cellular cleaning system that maintains metabolic balance under basal conditions and helps to ensure nutrient viability under stress conditions. It is also an important quality control mechanism that removes misfolded or aggregated proteins and mediates the turnover of damaged and obsolete organelles. In this regard, the idea that autophagy is a non-selective bulk process is outdated. It is now widely accepted that forms of selective autophagy are responsible for metabolic rewiring in response to cellular demand. Given its importance, autophagy plays an essential role during tumorigenesis as it sustains malignant cellular growth by acting as a coping-mechanisms for intracellular and environmental stress that occurs during malignant transformation. Cancer development is accompanied by the formation of a peculiar tumor microenvironment that is mainly characterized by hypoxia (oxygen < 2%) and low nutrient availability. Such conditions challenge cancer cells that must adapt their metabolism to survive. Here we review the regulation of autophagy and selective autophagy by hypoxia and the crosstalk with other stress response mechanisms, such as UPR. Finally, we discuss the emerging role of ER-phagy in sustaining cellular remodeling and quality control during stress conditions that drive tumorigenesis.

## 1 Introduction

The term autophagy, derived from the Greek “self-eating,” describes a catabolic process through which cytoplasmic cargos are delivered to lysosomes for degradation and recycling (He and Klionsky, 2009). Autophagy, together with the ubiquitin-proteasome system (UPS), are the two major protein degradative pathways in the cells and are essential for maintaining cellular proteostasis. However, while the UPS is mainly devoted to the rapid turnover of short-lived proteins, autophagy degrades long-lived proteins and organelles (Cohen-Kaplan et al., 2016). Under basal conditions, autophagy contributes to the metabolic balance of cells thus ensuring cell viability. Following lysosomal degradation, the breakdown products are released into the cytosol and recycled to generate energy (Kaur and Debnath, 2015). However, under stressful conditions such as nutrient starvation, hypoxia, genotoxic stress, and pathogen infection, autophagy is dramatically induced to preserve cellular homeostasis and to ensure proper quality control by removing misfolded or aggregated proteins, clearing damaged organelles, such as mitochondria and ER and eliminating intracellular pathogens (Tolkovsky, 2009; Galluzzi et al., 2017; Grumati et al., 2018; Reggio et al., 2020). Yet, insufficient, or excessive autophagy may lead to cell death. Thus, defective autophagy has been implicated in the pathogenesis of various diseases like cancer and neurodegenerative disorders (Mizushima et al., 2008). In cancer biology, autophagy is considered a double-edged sword since it can work both as a tumor suppressor and a tumor-promoting mechanism depending on the stage of cancer progression (Corcelle et al., 2009). It acts as a tumor-suppressive mechanism during cancer initiation and malignant transformation by removing damaged organelles, thereby limiting cell proliferation and genomic instability. On the other hand, autophagy is required for tumor progression and maintenance once malignant transformation is established, as cancer cells use autophagy for continuous supply of energy and nutrients (Corcelle et al., 2009).

It is now becoming clear that cancer cells are essentially characterized by a unique metabolic environment in which many stress-response pathways are activated and finely coordinated to promote tumor growth. The main driving force behind this metabolic rewiring is the establishment of a unique milieu within the tumor, known as tumor microenvironment (TME) (Yang, 2017). TME negatively affects normal cell physiology and facilitates cancerous transformation. Indeed, hyperproliferation of malignant cells causes loss of normal tissue architecture, accompanied by an abnormal vascularization, resulting in a dysfunctional distribution of nutrients, growth factors and oxygen within the tumor (Hanahan and Weinberg, 2011). Low oxygen availability, commonly referred to as hypoxia, is a hallmark of TME and forces cancer cells to adapt to a condition of constant stress. To cope with such stress, cancer cells activate a series of hypoxia-associated pathways comprised of HIFs, mTOR, and the unfolded protein response (UPR) which ultimately converge on autophagy (Wouters and Koritzinsky, 2008).

## 2 Overview of autophagy

### 2.1 Molecular basis of autophagy and selective autophagy

Different types of autophagy have been described in mammalian cells: macro-autophagy, micro-autophagy, and chaperone-mediated autophagy (CMA) (Parzych and Klionsky, 2014). The three types of autophagy differ in the specificity of the substrate, the delivery mechanism of the cargo, their regulation, and the conditions in which they are activated.

The CMA is a selective form of autophagy in which cytosolic proteins, equipped with a lysosomal targeting motif (KFER), are selectively recognized by the chaperone protein Hsp70, and delivered to the surface of the lysosome. At the lysosome the chaperone-protein complex interacts with the lysosomal associated membrane protein 2 (LAMP-2), promoting its translocation into lysosomal lumen for degradation (Cuervo and Wong, 2014).

In contrast to CMA, both micro- and macro-autophagy involve dynamic membrane rearrangement. During micro-autophagy, cytoplasmic components translocate into the lysosome through a direct invagination, protrusion, or septation of the lysosomal membrane (Li et al., 2012).

Macro-autophagy is the most well-characterized form of autophagy and, is generally referred to as autophagy. The main feature that characterizes and differentiates macro-autophagy - simply autophagy hereafter - from CMA and micro-autophagy is the delivery of cytosolic cargo proteins to lysosomes *via* a unique double-membrane vesicle structure called autophagosome (Feng et al., 2014)*.*

The formation of autophagosomes is a dynamic process that consists of several steps such as initiation, elongation, closure, and fusion with lysosomes. Each of these steps is finely regulated by a hierarchical interplay of autophagy-related proteins (ATGs) and signaling pathways that are differently activated upon a plethora of stimuli (Yang and Klionsky, 2010).

The initial step of autophagy is the surrounding and sequestering of cytoplasmic cargo and organelles within an isolation membrane called phagophore. This steps typically occurs in a specific structure, closely associated with the ER, called omegasome (Axe et al., 2008). In particular, upon autophagy induction, the Unc-51-like kinase 1/2 (ULK1/2), consisting of the serine/threonine kinase ULK1/2, ATG13, FIP200 (focal adhesion kinase family interacting protein of 200 kDa), and ATG101, translocates to the omegasome and regulates the recruitment of a second complex, the PI3KC3 (class III phosphatidylinositol 3-kinase) complex I, consisting of the VPS34, VPS15, Beclin-1and several binding proteins such as AMBRA1 (Di Bartolomeo et al., 2010; Nazio et al., 2013). The PI3KC3 complex I produces phosphatidylinositol 3-phosphate (PI3P) that acts as a platform for autophagosome biogenesis (Axe et al., 2008).

The main players in the next step are the two ubiquitin-like proteins, ATG12 and ATG8/LC3, that cooperate with their conjugation system to sustain the elongation of the phagophore membrane. ATG12 is conjugated to ATG5 to form the ATG12-ATG5 complex in a reaction mediated by ATG7 and ATG10 (E1 and E2-like enzymes, respectively). Then, the ATG12–ATG5 non-covalently interacts with ATG16L, which oligomerizes to form a large multimeric complex called the ATG16L complex. At this point, ATG5–ATG12–ATG16L complex promotes ATG3-mediated conjugation of activated LC3/GABARAP family members to the carboxyl glycine of phosphatidylethanolamine (PE) that is integrated into the growing phagophore (Kabeya et al., 2000; Nath et al., 2014). LC3 and its family members then mediate membrane tethering and hemi fusion, a function which is crucial for the expansion and fusion of autophagosomes with lysosomes (Nakatogawa et al., 2007). Elongation of phagophore requires membrane input from other organelles and ATG9 is the best candidate for this role. ATG9 is the only transmembrane ATG protein, identified thus far, that assists the growth of autophagic membranes by a poorly defined mechanism that may involve the trafficking of ATG9-containing vesicles between the trans-Golgi network, plasma membrane, recycling endosomes (REs), and autophagic membranes (Feng and Klionsky, 2017). Once the expanding ends of the phagophore fuse, the autophagosome is formed. At this point, newly formed autophagosomes undergo a maturation process. They fuse with early and late endosomes to form intermediate structures, called amphisomes, which, ultimately, fuse with lysosomes (He and Klionsky, 2009).

Autophagy has long been considered a non-selective bulk degradation process in which cytoplasmic cargo is randomly encapsulated in nascent autophagosomes. However, many forms of selective autophagy, which lead to the degradation of specific organelles, proteins and pathogens, have been described (Tolkovsky, 2009; Galluzzi et al., 2017; Grumati et al., 2018; Reggio et al., 2020).

Selective forms of autophagy share the same structural and molecular features as bulk autophagy, but are mediated by specific receptors (Stolz et al., 2014). Selective autophagy receptors, display exclusive binding regions which allow them to act as a bridge between their cargo and the autophagic machinery through the interaction with LC3/GABARAP family members (Johansen and Lamark, 2011; Johansen and Lamark, 2020; Gatica et al., 2018). These domains, known as AIM, LIR, or GIM (i.e., Atg8-interacting motif, LC3-interacting region, and GABARAP-interaction motif) are usually characterized by sequence resembling [W/F/Y] XX [L/V/I] where X can be any amino acid (Birgisdottir et al., 2013; Johansen and Lamark, 2020). Historically, the first autophagic receptor with a LIR consensus sequence to be identified was p62/SQSTM1. In addition to the LC3/GABARAP family, p62 can also bind ubiquitin (Ub) *via* a C-terminal UBA domain, thus allowing the degradation of ubiquitinated cargo by selective autophagy (Kirkin et al., 2009).

### 2.2 Regulation of autophagy

Given its importance, autophagy machinery is regulated by a plethora of signaling pathways. The principal player is the mammalian Target of Rapamycin (mTOR) (the mammalian ortholog of yeast TOR), which is a signaling control hub downstream of nutrients, growth receptors, hypoxia and ATP levels (Wullschleger et al., 2006). mTOR is a Serine/Threonine kinase that belongs to the phosphatidylinositol kinase-related kinase (PIKK) family (Abraham, 2004) and was first described as the physiological target of the immunosuppressant drug rapamycin (Sabers et al., 1995). In mammals, mTOR is sensitive to amino acids and glucose levels which, under normal conditions, keep mTOR active. Under conditions of nutrient depletion mTOR activity is inhibited, thus leading to the induction of autophagy (Jung et al., 2010). The mTOR pathway is composed of two functional complexes, that are differentially involved in autophagy modulation: the rapamycin-sensitive mTOR complex 1 (mTORC1), and the mTOR complex 2 (mTORC2) that is unaffected by rapamycin (Laplante and Sabatini, 2009). Under optimal growth conditions, mTORC1 directly inhibits autophagy through hyperphosphorylation of ATG13 and phosphorylation of ULK1 (Ser638 and Ser758). These modifications prevent the interaction between ATG13 and ULK1 and the formation of the initiation complex ULK1-ATG13-FIP200-ATG101 (Hosokawa et al., 2009). Nutrient deprivation, or rapamycin treatment, inactivates mTORC1 leading to the dephosphorylation of ATG13 and the formation of the initiation complex which translocates to the autophagy initiation sites and regulates the recruitment of the others ATG proteins (Jung et al., 2009). Inhibition of mTORC1 is associated with reduced phosphorylation of the ribosomal protein S6 kinase (also known as p70S6K) and the translation initiation factor 4E binding proteins-1 (4E-BP1), intended to slow down protein synthesis and meet the changing nutrient needs of the cells (Ma and Blenis, 2009).

As part of the energy-sensing cascade, mTORC1 senses changes in the extracellular energy state *via* AMP-dependent protein kinase (AMPK). Once activated, AMPK stimulates catabolic processes, ATP generating pathways and inhibits anabolic processes such as the synthesis of lipids, carbohydrates, and proteins to assure cell survival (Villanueva-Paz et al., 2016). In accordance with its physiological role, AMPK is linked to the regulation of autophagy. In particular, AMPK plays a dual role in activating autophagy by 1) inactivating mTORC1 and 2) directly activating ULK1, both *via* phosphorylation (Kim et al., 2011). Since mTORC1 is a key regulator of autophagy, one of the mechanisms of AMPK-dependent induction of autophagy is through mTORC1 inhibition. AMPK inhibits mTORC1 1) directly, by phosphorylating RAPTOR (Gwinn et al., 2008) and 2) indirectly, by phosphorylating Tuberous Sclerosis Complex 2 (TSC2) (Inoki et al., 2003). Moreover, AMPK can also activate autophagy *via* ULK1 phosphorylation. Under glucose starvation, AMPK directly phosphorylates of ULK1 at Ser317 and Ser777. Under nutrient rich conditions, on the other hand, active mTORC1 prevents ULK1 activation through the phosphorylation of Ser757, which disrupts the interaction between AMPK and ULK1 (Kim et al., 2011). Besides its role as a nutrient sensor, mTOR integrates numerous upstream stimuli including growth-factor signals (insulin or IGF-1), Toll-like receptor (TLR) ligands and cytokines, all of which converge on the tuberous sclerosis complex 1 and 2 (TSC1 and TSC2), the principle upstream inhibitor of mTOR. TSC2 forms a functional complex with TSC1 to inhibit the two key regulators of mTOR mediated translation: S6K and 4EBP1 (Huang and Manning, 2008).

## 3 Autophagy and cancer

As a central pathway involved in cellular metabolism, autophagy has been largely implicated in cancer biology. However, its role in cancer is complex since it can act as a tumor suppressor as well as a tumor-promoting mechanism (Corcelle et al., 2009; Kimmelman, 2011). Nowadays, is widely accepted that the role of autophagy in cancer is dynamic. Indeed, during the early stages of malignant transformation autophagy works as a tumor-suppressive mechanism limiting cellular proliferation by removing toxic proteins and organelles. Thus, preventing the accumulation of chronic cellular damage that could favor malignant transformation. In contrast, once malignant transformation has occurred, autophagy sustains cancer cell growth by supplying building blocks for macromolecule biosynthesis (Kimmelman, 2011).

Direct evidence for the role autophagy plays in tumor suppression come from mouse genetic studies employing Atg7, Atg5, and Beclin-1 knockout animals. The absence of these genes impairs autophagy flux and promotes tumor initiation (Qu et al., 2003; Takamura et al., 2011). For instance, the Beclin-1 locus is deleted in up to 75% of ovarian cancers and in up to 50%–70% of breast cancers (Liang et al., 1999). However, an important observation is that all of these tumors remain benign, indicating that even though autophagy depletion contributes to tumor initiation, functional autophagy machinery is required for tumor progression to a malignant stage (Takamura et al., 2011).

Additionally, the accumulation of p62, as a result of autophagy inhibition, is important in the promotion of tumorigenesis through a variety of mechanisms, including deregulation of NF-κB signaling, accumulation of ROS and increased DNA damage (Mathew et al., 2009). In particular, the accumulation of p62, in autophagy-deficient cells, inhibits the degradation of p53 and β-Catenin by sequestering ubiquitinated proteins and preventing their proteasomal degradation (Korolchuk et al., 2009).

On the other hand, other evidence suggest that autophagy plays a protective role in cancer cells and promotes tumor growth in advanced cancers (Luo et al., 2016; Liu et al., 2018). Indeed, rapidly developing cancer cells require cellular building blocks to support energy production. In accordance with this, a high basal level of autophagy was found in Ras mutated cancer cells (Su, 2018; Zhu et al., 2019). Moreover, even in the presence of copious nutrients, human cell lines with mutations in H-Ras or K-Ras, have elevated basal levels of autophagy (Guo et al., 2011).

## 4 Stress responses in the tumor microenvironment that converge on autophagy

### 4.1 Hypoxia

Regions with very low oxygen levels are found in many solid tumors and likely occur because of inadequate vascular formation due to the uncontrolled proliferation of malignant cells. Those regions where oxygen concentration is <2%, are named the hypoxic zone and represent one of the hallmark of cancer (Hanahan and Weinberg, 2011).

**FIGURE 1:**
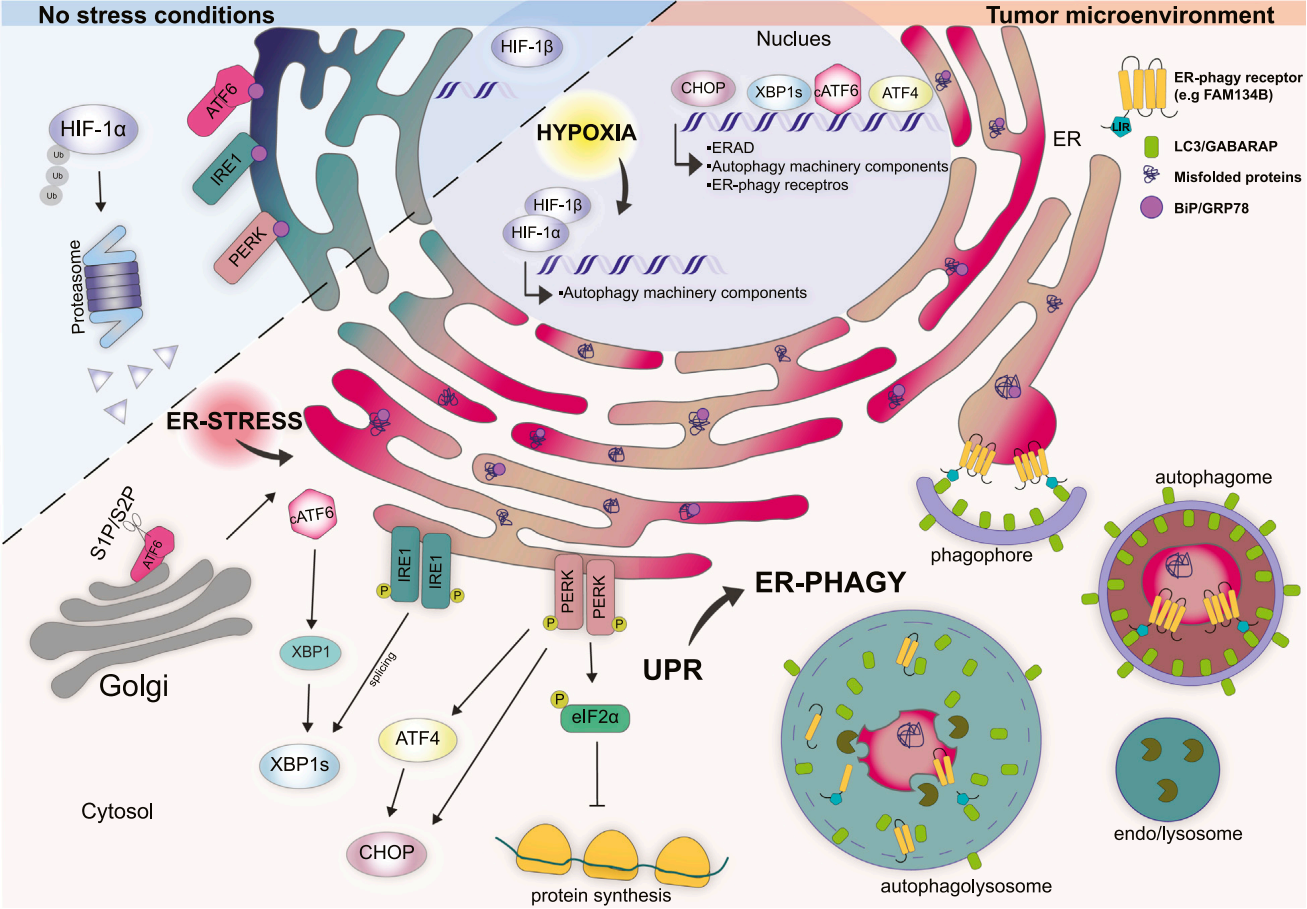
Cellular response mechanisms in non-stress conditions vs. tumor microenvironment. Under physiological conditions, HIF-1α is localized in the cytosol and rapidly degraded by the proteasome. The UPR stressors ATF6, IRE1, and PERK are kept inactive by the binding to the ER chaperone BiP/GRP78. Tumor microenvironment is characterized by hypoxia which induces the translocation of HIF-1α into the nucleus where it forms a stable complex with HIF-1β. HIF-1α/β complex regulates the transcription of several genes involved in the function and regulation of autophagy machinery. Hypoxia impairs the folding capacity of the ER and induces a rapid accumulation of misfolded proteins thus determining ER stress. Under ER stress conditions BiP/GRP78 bind misfolded proteins and dissociate from the UPR sensors. ATF6 translocates to the Golgi where is cleaved by S1P and S2P proteases that generate cytosolic transcription factor cATF6. cATF6 interact con XBP1 and regulates the transcription of genes involved in protein folding, trafficking and ERAD. Dimerization and phosphorylation of IRE1 activate the splicing of XBP1. PERK activation induces the phosphorylation of eIF2α preventing mRNA translation. Moreover, PERK induces the transcription factors ATF4 and CHOP. In the nucleus CHOP, ATF4, and XBP1s coordinate the transcription of several genes involved in the UPR, ERAD, and autophagy-related genes including some ER-phagy receptor genes. Together hypoxia, ER stress and UPR trigger ER-phagy to restore ER homeostasis.

The main transcription factor that regulates hypoxic response is HIF-1. HIF-1 is a heterodimeric complex consisting of the hypoxia-induced subunit HIF-1α and the constitutively expressed subunit HIF-1β that transcriptionally regulates the expression of several genes (Semenza, 2010). Because HIF-1β is constitutively expressed, HIF-1α, which contains an oxygen-dependent degradation domain (ODDD) and is tightly regulated by oxygen, is considered the major regulatory subunit of the HIF-1 complex (Semenza, 2007). Under normoxia, HIF-1α protein is rapidly degraded, resulting in minimal transcriptional activity of the HIF-1 complex. When cells are subjected to hypoxic conditions, HIF-1α is stabilized and translocates from the cytosol to the nucleus, where it interacts with HIF-1β to promote transcriptional activity (Semenza, 2007, 2010) (Figure 1). Autophagy is one of the pathways regulated by HIF-1. In particular, HIF-1 is involved in the regulation of key genes involved in the initiation and progression of autophagosome formation including BNIP3, BNIP3L/NIX, ATG7, ATG5, and ATG9A (Zhang and Ney, 2009; Gui et al., 2016; Abdul Rahim et al., 2017).

Bcl2/adenovirus E1B 19-kDa-interacting protein 3 (BNIP3) and BNIP3-like (BNIP3L), also known as NIX (BNIP3L/NIX) are two pro-apoptotic proteins that localize to the outer mitochondria membrane (OMM) and share several common features (Ney, 2015). Their expression is transcriptionally regulated by HIF-1α during hypoxia and both play an important role in the modulation of hypoxia induced-autophagy (Zhang and Ney, 2009). In particular, BNIP3/BNIP3L influence autophagy by modulating the Bcl2-Beclin-1 and BclXL-Beclin-1 complexes (Bellot et al., 2009). Under non-stressful conditions, both Bcl2 and BclXL are bound to Beclin-1 thus inhibiting phagophore formation. The upregulation of BNIP3/BNIP3L upon hypoxia induces the release of Beclin-1 from Bcl2 and BclXL which bind with higher affinity to BNIP3 and BNIP3L *via* their BH3 domain (Bellot et al., 2009). The free form of Beclin-1 is then able to interact with the VPS34 complex and induce autophagy (Bellot et al., 2009). Beyond their contribution to modulating general autophagy, BNIP3 and BNIP3L can also act as mitophagy receptors. Indeed, they harbor a LIR motif that is exposed to the cytoplasm, through which they interact with LC3/GABARAP and mediate clearance of mitochondria *via* selective autophagy (Novak et al., 2010; Hanna et al., 2012). The precise mechanism of BNIP3/BNIP3L induced-mitophagy is not completely clear, but it seems that several phosphorylation events are crucial for the function of these proteins as mitophagy receptors. In particular, the phosphorylation of BNIP3L at Ser81 is necessary for mitophagy induction under ischemic conditions (Yuan et al., 2017). Moreover, BNIP3 phosphorylation at Ser17 and Ser24 strongly increases its interaction with LC3, thus promoting mitophagy (Yuan et al., 2017). However, the precise mechanism by which these two receptors are modulated in response to hypoxia remains to be elucidated and warrants further investigation.

In addition to BNIP3/BNIP3L, FUNDC1 is another integral OMM protein containing a LIR domain that is implicated in hypoxia-induced mitophagy (Liu et al., 2012). Indeed, FUNDC1 mediated-mitophagy is appears to be specifically triggered under hypoxic conditions, since FUNDC1 knockdown in HeLa cells does not affect starvation-induced mitophagy, and only plays a moderate role in FCCP-induced mitophagy (Liu et al., 2012). From a mechanistic point of view, during normoxia FUNDC1 is phosphorylated by SRC kinase at Tyr18, and by casein kinase 2 (CK2) at Ser13, on its LIR domain. These phosphorylation events decrease FUNDC1’s binding affinity for LC3, thus preventing cargo recognition within the autophagosome (Liu et al., 2012; Kuang et al., 2016). On the contrary, upon hypoxia, both SRC and CK2 are inactivated and FUNDC1 phosphorylation is significantly reduced, thus allowing it to strongly interact with LC3B-II, resulting in the removal of damaged mitochondria by mitophagy. In particular, CK2 is phosphorylated under normoxia but dephosphorylated in response to hypoxia by the mitochondrial phosphatase PGAM5 (Chen et al., 2017). PGAM5 is normally inhibited by the interaction with BclXL which blocks its phosphatase activity. When cells are under hypoxic conditions, the degradation of BclXL induces PGAM5 activation which, in turn, dephosphorylates FUNDC1 at Ser13 (Wu et al., 2014). The inactivation of SRC under hypoxic conditions is mediated by a single phosphorylation event at Tyr416 (Ozkirimli and Post, 2006). As a consequence, phosphorylation at this site blocks FUNDC1 phosphorylation at Tyr18 (Ozkirimli and Post, 2006). This two-part system allows the fine-tuning of mitophagy during hypoxia.

### 4.2 ER stress

The ER is the largest organelle in the cell consisting of a large membrane network that originates from the nuclear envelope and spreads throughout the cytosol. Structurally, it can be divided into sheets and tubules. The sheets are covered by ribosomes and form the rough ER, which is the primary site of protein synthesis and folding. The tubules form smooth ER whose function is to synthesize lipids and steroids and buffer cytosolic calcium (Schwarz and Blower, 2016). Moreover, the ER has the important task of interacting with other organelles such as the Golgi apparatus, mitochondria, lysosomes, endosomes, and even the plasma membrane, at highly specialized membrane contact sites (Phillips and Voeltz, 2016). These contacts regulate organellar functions such as Ca^2+^ homeostasis, lipid composition, fission, trafficking, and participation in signal transduction events (Phillips and Voeltz, 2016).

Several cellular stressors, such as Ca^2+^ levels, redox imbalance or altered protein glycosylation, can disrupt ER homeostasis and lead to a condition known as ER stress. Low oxygen availability and glucose shortage, which characterize the tumor microenvironment, are well-characterized ER stress stimuli. Under such conditions, the ability of the ER to form disulfide bonds is impaired, with the consequent accumulation of unfolded/misfolded proteins in the ER lumen (Chipurupalli et al., 2019). To overcome this stressful condition, cells have developed an integrated system of stress-responsive pathways composed of the UPR, ERAD, and autophagy, to restore homeostasis and normal ER function.

The unfolded protein response (UPR) is triggered by the accumulation of unfolded/misfolded proteins with the aim to re-establish ER homeostasis (Corazzari et al., 2017). UPR makes use of three ER-transmembrane proteins that act as stressor sensors: the inositol-requiring enzyme 1α (IRE 1α), the pancreatic endoplasmic reticulum kinase (PERK), and the activating transcription factor 6 (ATF6) (Gardner et al., 2013) (Figure 1). Mechanistically, during non-stress conditions the activation of these sensors is inhibited by the binding with the chaperon protein BiP - binding immunoglobulin protein - (also known as GRP78 - 78 kDa glucose-regulated protein), which is the most represented ER chaperone (Bertolotti et al., 2000). Conversely, upon ER stress BiP dissociates from the ER stress sensor, due to its capacity to bind misfolded proteins with higher affinity, thus leading to the activation of the above-mentioned sensors and their downstream signaling (Figure 1) (Bertolotti et al., 2000).

PERK is a transmembrane type I protein characterize by a cytosolic serine/threonine kinase domain which is enriched at the mitochondria-associated ER membrane sites (MAMs) (Verfaillie et al., 2012). Upon ER stress, BiP dissociates from PERK thus inducing its activation through homodimerization and autophosphorylation (Bertolotti et al., 2000). The best-characterized substrate of activated PERK is the eukaryotic translation initiation factor 2α (eIF2α) (Harding et al., 1999). PERK-dependent phosphorylation of eIF2α, at Ser 51, attenuates protein synthesis by global inhibition of 5′ cap-dependent translation while selectively increasing the cap-independent translation of many mRNAs, such as ATF4 - activating transcription factor 4 (Harding et al., 2000; Scheuner et al., 2001). ATF4, in turn, induces the transcription factor C/EBP (CHOP) and several other genes involved in the regulation of autophagy, amino acid metabolism, antioxidant response, and even cell death (Figure 1) (Lu et al., 2004; Vattem and Wek, 2004; Liu et al., 2015).

IRE1α is the most evolutionarily conserved arm of the UPR and consists of a type I transmembrane protein equipped with a cytosolic Serine/Threonine kinase domain (Cox et al., 1993). Upon activation, which occurs because of oligomerization and autophosphorylation, IRE1α goes through a conformational change that activates its RNAase activity. Activation of IRE1α promotes the unusual splicing of XBP1 - X-box-binding protein - mRNA, thus generating a modified mRNA isoform, indicated as *XBP1s* (where “s” means spliced). The spliced mRNA encodes for a functional XBP1s protein that acts as a transcription factor and regulates the expression of genes involved in protein folding, trafficking as well as components of the ER-associated protein degradation program (ERAD) (Figure 1) (Yoshida et al., 2001).

ATF6 is a type II transmembrane protein that is characterized by a cAMP-responsive element-binding protein and an ATF basic leucine zipper domain. During ER stress ATF6 translocates to the Golgi apparatus where it is cleaved by S1P and S2P proteases (known as MBTPS1 and MBTPS2) generating a cytosolic transcription factor (Haze et al., 1999). Cleaved ATF6α forms a heterodimer with XPB1 and regulates the transcription of unspliced XBP1 and other genes involved in protein folding and degradation in the ER such as chaperones, foldases and ERAD components (Lee et al., 2002; Lee et al., 2003). The overall outcome, from the activation of these three branches of the UPR, is to slow down protein synthesis, enhance the ER’s post-translation modification capacity and increase the degradation of unfolded/misfolded proteins by the ERAD system and autophagy (Figure 1).

Considering its vital role in ER stress mitigation, it is not surprising that UPR activation is a hallmark of several human cancers. Indeed, the UPR enables cancer cell survival and enhances their ability to adapt to adverse environmental conditions (Romero-Ramirez et al., 2004; Fels and Koumenis, 2006). In support of this notion, accumulating evidence demonstrate that all the molecular actors of the UPR are involved in tumor growth in one way or another. For example, IRE1 signaling seems to be crucial for hepatocellular carcinoma (HCC) initiation, whereas PERK activation is essential in the later stage of tumor progression (Vandewynckel et al., 2015). Moreover, both BiP and XBP1 are implicated in sustaining tumor cell survival and in mediating the tumor cells’ response to glucose deprivation (Spiotto et al., 2010). Furthermore, all three UPR signaling pathways have been shown to be activate during prostate cancer progression (Liu et al., 2017).

#### 4.2.1 Crosstalk between UPR, Ca^2+^ signaling, and autophagy

Nowadays, it is widely accepted that UPR does not act alone to counteract ER stress but is rather closely associated with autophagy. Indeed, all three branches of the UPR positively modulate autophagy induction upon ER stress (Verfaillie et al., 2010).

IRE1 actively modulates ER stress induced autophagy. In particular, active IRE1 mediates mitogen-activated protein kinase 8 (MAPK8) activation which, in turn, induces autophagy (Ogata et al., 2006). MAPK8 is considered a major stress-regulated protein kinase that controls stress-induced autophagy and apoptosis (Xia et al., 1995; Ogata et al., 2006). Mechanistically, MAPK8 interacts with c-Jun N-terminal kinase (JNK) that phosphorylates the two autophagy inhibitory proteins, BcL-2 and BcL-XL, which dissociate from the key autophagy inducer Beclin-1, resulting in autophagy induction (Wei et al., 2008; Corazzari et al., 2015). In addition, the IRE1-XBP1s axis has also been involved in the induction of autophagy (Margariti et al., 2013). In particular, XBP1s directly binds the Beclin-1 promoter in the nucleus, thus enhancing autophagy induction *via* the transcriptional upregulation of Beclin-1 (Suzuki et al., 2009; Margariti et al., 2013).

In addition, the ectopic expression of aggregated polyglutamine proteins (poliQ) triggers autophagy in a PERK-dependent manner, further supporting the link between UPR and autophagy. In particular, genetic substitution of Ser51 with Ala on eIF2α, prevented polyQ protein clearance by autophagy, suggesting a prominent role for the PERK-eIF2α axis in the activation of autophagy in response to the accumulation of unfolded/aggregate proteins (Kouroku et al., 2007). PERK activation also results in the downstream expression of both ATF4 and CHOP. ATF4 induces autophagy by increasing the expression of ATG12 (Kouroku et al., 2007) and others important autophagy genes like MAP1LC3, BECN1, ATG3, ATG12, and ATG16L1 (B’chir et al., 2013), while CHOP positively regulates the expression of Tribbles Homolog 3 (TRB3) resulting in the downstream inhibition of the mTOR complex through the inhibition of AKT activity, thus further stimulating autophagy (Salazar et al., 2009).

It has been suggested that the activation of the ATF6 branch of UPR, also triggers autophagy. Indeed, upon ER stress cATF6 induces the expression of Death-Associated Protein Kinase 1 (DAPK1) (Kalvakolanu and Gade, 2012; Gade et al., 2014), which phosphorylates Beclin-1 and promotes its dissociation from its negative regulator Bcl2, thus promoting autophagosome formation. Moreover, cATF6 also indirectly regulates autophagy *via* CHOP and XBP1 (Zalckvar et al., 2009).

ER stress is often accompanied by the release of calcium into the cytosol. Acting as a second messenger, calcium activates several Ca^2+^-dependent signaling pathways, including autophagy. In particular, the release of Ca^2+^ from ER induced by thapsigargin, ionomicyn, and vitamin D analogues, activates the Ca^2+^/calmodulin-dependent kinase *β*, which in turn activates AMPK and inhibits mTOR, thereby inducing autophagy (Høyer-Hansen et al., 2007).

Moreover, it has been shown that the activation of protein kinase C theta (PRKCQ/PKCθ) in response to ER stress can modulate autophagy in a Ca^2+^-dependent manner (Sakaki and Kaufman, 2008). Indeed, Ca^2+^ is an essential component of PRKCQ activation under ER stress conditions, which is supported by the observation that cells treated with the intracellular Ca^2+^ chelator BAPTA-AM deactivate PRKCQ with a consequent blockage of autophagy flux (Sakaki et al., 2008). Altogether, these reports support a crucial function of cytosolic Ca^2+^ mobilization, and modulation of Ca^2+^-related pathways upon ER stress in autophagy induction.

In this scenario, emerging data indicate an important role for mitochondria associated ER-membranes (MAMs). MAMs are flexible ER membrane-derived structures in which ER and mitochondria are physically and functionally connected (Csordás et al., 2006). MAMs serve as structural microdomains that allow the transfer of Ca^2+^ between the ER and mitochondria to regulate mitochondria bioenergetics and likely contribute to enhancing mitochondrial ATP production to satisfy increased energy demands resulting from stress. Consistently, alterations in the interaction between ER and mitochondria leads to the disruption of Ca^2+^ transfer between the two organelles, and to ER stress (Barazzuol et al., 2021). Moreover, MAM-mediated mitochondrial Ca^2+^ overload leads to mitochondrial depolarization and eventually triggers cell death (Walter and Hajnóczky, 2005).

The cross talk between ER and mitochondria is modulated in different ways and by numerous players. Among them, Mitofusin 2 (MFN2), a dynamin-related GTPase localized to the surface of both ER and mitochondria. The homologous interaction between ER-associated MFN2 and mitochondrial MFN2 facilitates interorganelle tethering (de Brito and Scorrano, 2008). Ablation of MFN2 results in an increased distance between ER and mitochondria and a disturbed Ca^2+^ transfer between the two organelles (de Brito and Scorrano, 2008). MFN2 is an important regulator of UPR and mitochondrial functions. Indeed, MFN2 interacts with PERK, at MAMs, to suppress its activation under normal conditions. Loss of function of MFN2 causes an increase in the production of reactive oxygen species (ROS), mitochondrial calcium overload, and impaired mitochondrial morphology through the sustained activation of PERK. Consistently, PERK inhibition rescues mitochondrial integrity in MFN2 mutant cells, thus identifying the MFN2-PERK axis as an important regulator of mitochondrial processes (Muñoz et al., 2013). Accordingly, PERK is also crucial for regulating MAMs integrity (Verfaillie et al., 2012). These data point out the important role of UPR and MAMs in regulating ER and mitochondrial dynamics and maintaining cellular homeostasis.

#### 4.2.2 ER stress, UPR, and autophagy in immune-modulation and inflammation

A growing body of evidence suggests that, in cancer cells, chronic activation of the ER stress response influences malignant progression by altering the function of the immune cells that coexist in the tumor microenvironment. The immune response is a key element implicated in cancer development. During early stages of tumorigenesis, the immune system is able to recognize and suppress tumor cells that express neoantigens in major histocompatibility complex class I (MHC-I) molecules, through natural killer (NK) cells and cytotoxic lymphocytes (CTL) (Dunn et al., 2002). Early evidence suggests that the induction of ER stress and activation of the UPR may inhibit the surface expression of MCH-I molecules *via* XBP1 and ATF6 overexpression (de Almeida et al., 2007). Moreover, increased ER stress triggered by glucose withdrawal in EL4 lymphoma cells, caused eIF2α-mediated inhibition of protein synthesis with a consequent impairment of MHC-I peptide presentation (Granados et al., 2009). Disruption of MHC-I antigen presentation has also been linked to the XBP1 branch of the UPR. In epithelial cells treated with the proteasome inhibitor ALLN and tunicamycin, to induce ER stress, XBP1-dependent induction of miR-346 negatively regulated the expression of the ER transporter involved in antigen processing 1 (TAP1) that is implicated in proper ER peptide influx and MCH-I antigen presentation (Bartoszewski et al., 2011). However, whether chronic induction of UPR in cancer cells facilitates immune evasion toward the above-mentioned mechanism needs to be further examined. Of note, some evidence demonstrate that autophagy may also play a role in the immune evasion of cancer cells. Autophagy promotes the degradation of MHC-I molecules with a consequent reduction in their surface expression in pancreatic ductal adenocarcinoma (Yamamoto et al., 2020). In particular, MHC-I molecules are targeted for selective autophagic degradation mediated by NBR1 and pharmacological and genetic inhibition of autophagy increases the surface expression of those molecules, thus restoring the susceptibility of pancreatic cells to elimination by CTL cells (Yamamoto et al., 2020).

ER stress can influence the immune response within the TME through the so-called transmissible ER stress - TERS. Soluble factors released from prostate, lung or melanoma cancer cells undergoing ER stress, can induce similar ER stress and UPR activation in bone marrow-derived myeloid cells, that is accompanied by the induction of pro-tumorigenic and immunosuppressive functions (Mahadevan and Zanetti, 2011). Additional studies further confirmed that TERS facilitates the communication between ER stressed cancers cells and other cancer cells or tumor-infiltrating immune cells such as tumor-associated macrophages (TAMs), (Rodvold et al., 2017) and that this phenomena reduced antigen processing and presentation and diminished T cell proliferation (Mahadevan et al., 2012).

Lastly, ER stress has been involved in the modulation of the inflammatory response within the TME. Indeed, all three UPR-branches induce the translocation of NF-κB to the nucleus with consequent inflammatory gene transcription, albeit *via* different mechanisms. PERK leads to NF-κB activation *via* a peculiar mechanism in which translational inhibition reduces the IκB/NF-κB ratio, therefore allowing the nuclear translocation of NF-κB and the transcription of inflammatory genes (Deng et al., 2004). Upon activation, IRE1α forms a complex with TNF-αR–associated factor 2 (TRAF2) at its cytosolic domain, that directly mediates IκB phosphorylation *via* IκB kinase (IKK), thus leading to NF-κB activation (Hu et al., 2006). Moreover, ATF6 was shown to participate in NF-κB activation in an AKT-dependent manner (Yamazaki et al., 2009).

These data support the idea that the ER stress response is a key regulator of cellular inflammation. However, a mechanistic link between ER stress induced autophagy, immunomodulation, and chronic inflammation within the tumor microenvironment remains to be better characterized.

## 5 ER-phagy

The term ER-phagy refers to the selective degradation of a discrete portion of ER within an autophagolysosome (Grumati et al., 2018). Given its complexity, the ER is a highly dynamic organelle that undergoes continuous remodeling to accommodate the changing demands in cellular protein homeostasis. The selective autophagic degradation of the ER, known as ER-phagy, substantially contributes to the ER remodeling process (Wilkinson, 2019; Gubas and Dikic, 2022).

ER degradation can occur either *via* micro- or macro-ER-phagy. Micro-ER-phagy occurs through direct invagination of ER portions into the endo-lysosomes, whereas macro-ER-phagy relies on a subset of specific cargo receptors and depends on the core molecular autophagy machinery (Reggiori and Molinari, 2022). Mechanistically, these receptors promote ER fragmentation and engage luminal, membrane-bound, and cytosolic factors that drive lysosomal clearance of select ER domains along with their content (Grumati et al., 2018).

### 5.1 ER-phagy receptors

To date, eight ER-resident proteins have been characterized as ER-phagy receptors in mammals: the family with sequence similarity 134 members (FAM134A, B, C) (Khaminets et al., 2015; Reggio et al., 2021), Reticulon 3 (RTN3) (Grumati et al., 2017), Translocation protein SEC62 (SEC62) (Fumagalli et al., 2016), Cell-Cycle Progression Gene 1 (CCPG1) (Smith et al., 2018), Atlastin 3 (ATL3) (Chen et al., 2019), and Testis-Expressed protein 264 (TEX264) (An et al., 2019; Chino et al., 2019). As selective autophagy receptors, they harbor a functional LIR domain, that allows their binding with the LC3/GABARAP, and are degraded inside lysosomes along with the ER.

**FIGURE 2:**
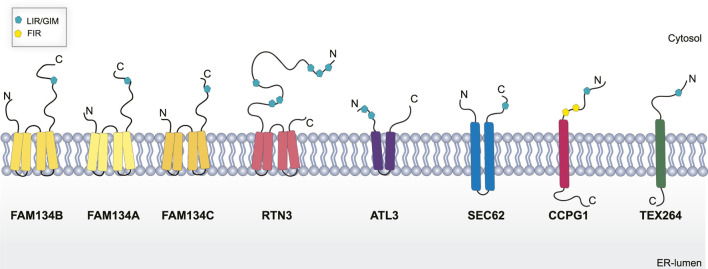
Schematic representation of ER-phagy receptors. Abbreviations: LIR/GIR, LC3/GABARAP interacting region; FIR, FIP200 interacting region; C, C terminal domain; N, N-terminal domain.

FAM134B, also known as RETREG1 or JK1, was the first ER-phagy receptor to be identified (Khaminets et al., 2015). Structurally, it is an intra-membrane protein that localizes to ER sheets and has a LIR domain at the C-terminal end (Figure 2) (Khaminets et al., 2015). Cells depleted of FAM134B show a substantial increase in ER volume and its deletion *in vivo* causes severe sensory neuropathy (Kurth et al., 2009; Khaminets et al., 2015). Conversely, overexpression of FAM134B causes an increased ER fragmentation and re-shuffling of ER membranes into autophagosomes (Khaminets et al., 2015). This indicates the fundamental role of FAM134B in maintaining ER shape and homeostasis. On the contrary, FAM134A and FAM134C do not have a similar role under basal conditions but are mainly activated upon stress (Reggio et al., 2021).

FAM134B was first described as an oncogene in esophageal squamous cell carcinoma (ESCC) (Tang et al., 2001). However, it is now widely accepted that it has a dual role in cancer, acting as a tumor-promoter in ESCC and in hepatocellular carcinoma (HCC), or a tumor suppressor in colorectal cancer (CRC) and breast cancer (BC) (Tang et al., 2007; Dai et al., 2017; Islam et al., 2017; Zhang et al., 2019; Table 1). The precise mechanisms behind these dual roles are far from being understood, even though several lines of evidence indicate that it may regulate cancer cell death and apoptosis through its role as an ER-phagy receptor (Mo et al., 2020).

**TABLE 1:** ER-phagy receptors in different cancer subset.

ER-phagy receptor	Type of cancer	Function	Reference
FAM134B	Colorectal and breast cancer	Tumor suppressor	Dai et al. (2017); Islam et al. (2017)
	Hepatocarcinoma and esophageal squamous cell carcinoma	Oncogene	Tang et al. (2007); Zhang et al. (2019)
RTNL3	Hepatocarcinoma	Cell growth arrest	Song et al. (2021)
SEC62	Lung, prostate, tyroid cancer	Increased invasion and metastasis	Bergmann et al. (2017)
	Colorectal cancer	Increased stemness properties *via* Wnt/β-Catenin pathway modulation	Liu et al. (2021)
CCPG1	Pancreatic cancer	Depolarization of acinar cells	Smith et al. (2018)
TEX264	Colorectal cancer	Tumor suppressor	Liu and Liu (2015)

RTN3L is devoted to the selective degradation of ER tubules (Grumati et al., 2017). It is part of the RTNs family which includes RTN1, RTN2, RTN3, and RTN4. Each of them contains a highly homologous RHD domain but they significantly differ in their N-terminal domain (Grumati et al., 2017), have several isoforms with different lengths (Yang and Strittmatter, 2007). The only member of this family characterized as an ER-phagy receptor is the long isoform of RTN3 (RTN3L). It harbors six functional LIR domains in the N terminal portion of the protein (Figure 2), and forms dimers to fragment ER tubules into discrete portions to be delivered lysosomes (Grumati et al., 2017). RTN3 has also been implicated in cancer. It acts as a tumor suppressor in hepatocellular carcinoma (HCC) inducing cell growth arrest, both *in vitro* and *in vivo*, by facilitating p53 phosphorylation, at Ser392, *via* Chk2 (Song et al., 2021; Table 1). However, the precise role of RTN3 in cancer needs to be further elucidated.

ATL3 is another ER-phagy receptor belonging to the Atlastin protein family, which includes ATL1, and ATL2 (Rismanchi et al., 2008). These are dynamin-like proteins that favor ER membrane fusion through a conformational change driven by GTP hydrolysis (Bian et al., 2011). ALT3 has two GIM motifs (Figure 2) that specifically interact with GABARAP and promote selective degradation of ER during starvation (Chen et al., 2019). Interestingly, mutations in ALT3 are associated with hereditary sensory and autonomic neuropathy type I (HSAN I). Mutations at Y192C and P338R affect ATL3 binding avidity to GABARAP, therefore, hampering its function as an ER-phagy receptor (Chen et al., 2019).

SEC62 together with SEC61 and SEC63 form the multiprotein translocon complex which imports newly nascent polypeptides from ribosomes into the ER (Linxweiler et al., 2017); however, SEC62 is the only one among them to be linked to ER-phagy. As an ER-phagy receptor, it is equipped with a LIR domain at the C-terminal (Figure 2). SEC62 mediated ER-phagy is activated in eukaryotic cells recovering from an ER stress inducing condition (Fumagalli et al., 2016). Indeed, mammalian cells tend to expand their ER surface in response to stress. Therefore, SEC62-mediate ER-phagy aims to reshape the ER to a pre-stress state, thus it is also called recov-ER-phagy (Fumagalli et al., 2016).

According to these features, in non-small cell lung, thyroid, and prostate cancer, upregulation of SEC62 confers tolerance to ER stress and contributes to invasion and metastasis (Bergmann et al., 2017). Moreover, in colorectal cancer, increased expression of SEC62 enhances stemness properties by modulating the Wnt/β-Catenin pathway, and this is associated with poor prognosis in patients (Liu et al., 2021; Table 1).

CCPG1 is a transmembrane ER-phagy receptor that contains a LIR domain and a FIP200 interacting region (FIR) (Figure 2) indicating a potential role of this receptor in the initiation step of autophagosome formation (Smith et al., 2018). Of note, CCPG1 is upregulated in response to UPR stress induced by DTT, tunicamycin and thapsigargin. Moreover, in these conditions, CCPG1-mediated ER-phagy depends on the binding of both ATG8 and FIP200 (Smith et al., 2018). Consistent with its role in proteostasis, CCPG1 knockout mice display depolarization of acinar cells of the exocrine pancreas that was accompanied by the accumulation of ER-produced secretory proteins (Smith et al., 2018; Table 1).

Finally, TEX264 is a single-pass transmembrane protein (Figure 2) individuated as an ER-phagy receptor in two independent proteomics-based studies (An et al., 2019; Chino et al., 2019). TEX264 localizes, in punctate structures, at the three-way junctions of the ER. Moreover, through a proximity labeling approach, other proteins involved in autophagosome formation, such as VPS34 complex proteins and WIPI2, have been detected in proximity with TEX264. This finding led to the hypothesis that TEX264 acts as a zipper-like helper allowing the ER membrane to enter into close contact with the phagophore membrane through a *trans* interaction (An et al., 2019). TEX264 is a marker protein for colorectal cancer, but its precise contribution in tumor development need to be further elucidated (Liu and Liu, 2015; Table 1).

### 5.2 ER-phagy as stress response: Crosstalk between UPR and ER-phagy

Autophagy induction triggered by ER stress, has a dual role in re-establishing general cellular homeostasis and to restoring internal ER-homeostasis by reestablishing its physiological size. To do so, the UPR induces the selective degradation of ER membranes and luminal proteins *via* ER-phagy (Molinari, 2021).

The first evidence that supported the idea of UPR-induced ER remodeling, comes from study from Bernales and colleagues. They treated wild-type cells with dithiothreitol to induce UPR, and then carried out an ultrastructural analysis of the ER. They found that folding stress significantly increased the volume of ER in yeast and that ER expansion was followed by the appearance of large double membrane vesicles filled with stacked membrane cisternae in many cells. They examined flash-frozen/freeze-substituted sections stained with osmium and found the delimiting outer membranes were densely decorated with ribosomes, suggesting that those were ER-derived membranes. They therefore referred to these vesicles as ER-containing autophagosomes, or ERAs. To further confirm this observation, they performed immunogold labeling of an ER marker protein, and observed selective labeling of clearly identifiable ER structures, as well as selective labeling of ERAs. Therefore both the sequestered and the sequestering membranes come from the ER, indicating that the formation of ERAs involves an engulfment of the ER by itself, and this process is termed “ER-phagy” (Bernales et al., 2006).

ER turnover *via* autophagy can be elicited by several stimuli including nutrient deprivation and chemicals agents (e.g., thapsigargin and dithiothreitol -DTT-) that are also able to induce general autophagy, ER stress and UPR. In some cases, these pleiotropic signals may increase the level of ER-phagy receptors. FAM134B was shown to be transcriptionally regulated by TFEB/TFE3 transcription factors, in MEFs and HeLa cells upon prolonged starvation (Cinque et al., 2020). Activation of the TFEB/TFE3 axis also increases FAM134B expression in chondrocytes exposed to fibroblast growth factor 18 (FGF18) (Cinque et al., 2020). In glioblastoma cells, FAM134B and TEX264 transcription is highly induced upon treatment with loperamide, a compound that induces ER stress resulting in autophagy-induced cell death (Liao et al., 2019; Koenig et al., 2020; Zielke et al., 2021). SEC62 expression and the following SEC62-mediated ER-phagy is enhanced by globular adiponectin treatment of cardiomyocytes subjected to chronic intermittent hypoxia, which protects them from ER stress-induced cell death (Zhang et al., 2020). CCPG1 expression is increased in cells treated with DTT, tunicamycin, or thapsigargin (Smith et al., 2018). In particular, evidence suggests that transcriptional regulation of this ER-phagy receptor depends on PERK (Adamson et al., 2016). These data support the idea that ER-phagy acts as a stress response mechanism that works in concert with the UPR to resolve the stressful condition. Consistently, MCF-7 cells (breast cancer) survive hypoxia induced ER stress by promoting FAM134B mediated ER-phagy (Chipurupalli et al., 2022).

As part of the recovery process, ER-phagy ensures proper ER fitness by eliminating excess ER membranes, created in response to stress, and may contribute to the elimination of protein aggregates. FAM134B binds misfolded procollagen with the aid of the ER chaperone Calnexin (CANX), which acts as a co-receptor. In particular, CANX recognizes and binds misfolded procollagen in the ER lumen and interacts with FAM134B which drives the autophagic degradation of both procollagen and CANX (Forrester et al., 2019). However, it is not clear whether ER-phagy contributes clearing out of UPR substrates when the UPR is defective.

Other observations further support the crosstalk between the UPR and ER-phagy. In particular: 1) chemical inducers of the UPR also trigger ER-phagy (Bernales et al., 2006; Ogata et al., 2006; Moretti et al., 2017; Liu et al., 2019), 2) ER-phagy protects from cell death upon the induction of the UPR (Ogata et al., 2006; Zhao et al., 2020), 3) ER-phagy receptors such as FAM134B, TEX264 (Zielke et al., 2021), and CCPG1 are induced upon the activation of the UPR and promote ER turnover (Smith et al., 2018) and 4) ER-phagy receptors, such as SEC62, are activated during recovery from ER stress and play a crucial role in resuming the physiological size and function of the ER (Fumagalli et al., 2016).

Ultimately, it can be affirmed that, by working in a coordinated manner, UPR and ER-phagy ensure ER plasticity, which is essential for a proper response to the metabolic changes of the cell. However, the mechanistic details of this relationship have yet to be fully understood (Figure 1).

## 6 Conclusion

In response to the stressful conditions within the tumor microenvironment, cancer cells must adapt their metabolism by activating a series of metabolic pathways that ensure their survival. Hypoxia is a stressful condition that drives such metabolic changes. It triggers autophagy and UPR which, in turn, sustain the induction of autophagy, creating a feedback loop of signaling pathways that are differentially modulated. Under stress conditions, both bulk and selective autophagy are actively regulated implicating ER-phagy as an important piece of this complicated puzzle. Although a direct link between ER-phagy and cancer progression is still missing, several lines of evidence support this hypothesis. Indeed, operating in concert with the UPR, and being itself induced upon hypoxia, ER-phagy may contribute to cancer cell homeostasis by maintaining ER fitness. In this scenario, hypoxia acts as an ER stress inducer while UPR and ER-phagy are prompted to resolve the stress and promote cell survival. In a tumorigenic environment this is favoring cancer cell adaptation and proliferation. However, the ER-phagy field is still in its infancy and much more needs to be understood. Although the number of ER-phagy receptors identified and characterized in recent years has exponentially increased, very little is known regarding the regulatory mechanisms that lead to their activation. Understanding these pathways will help uncover the role of ER-phagy in cancer progression, paving the way to the discovery of new druggable targets.
